# The extracellular vesicle-based treatment: a developing strategy for periodontal diseases

**DOI:** 10.3389/fimmu.2025.1480292

**Published:** 2025-05-29

**Authors:** Xuan Zhang, Hanyu Gao, Li Lin

**Affiliations:** Department of Periodontics, School and Hospital of Stomatology, China Medical University, Shenyang, China

**Keywords:** extracellular vesicles, periodontitis, periodontitis treatment, periodontal regeneration, extracellular vesicle-based strategies

## Abstract

Periodontitis, a chronic inflammatory disease, is characterized by irreversible bone resorption, persistent attachment loss, and even ultimate tooth loss. The individual quality of life and the public burden of health are greatly impacted by the destruction of periodontal tissue and the lack of effective treatment. Exploration into how the inflammatory and damaged structure changes into the healthy and regenerative tissue is one of the most fascinating subjects in this field. A novel approach is the application of a nanoparticle, extracellular vesicle, which originates from natural or engineered cells. To further exploit its full potential, scientists have conducted massive studies into its biological and functional mechanisms. This review provides an overview of current extracellular vesicle-based treatments on periodontal diseases. It begins with summarizing the history of periodontal regeneration. Then, this article takes a general overview of extracellular vesicle’s biological characteristics, structures and current achievements. After that, we discussed extracellular vesicle’s functions in periodontium. In addition, this study also embraces a diverse range of extracellular vesicle-based strategies, a general workflow for EVs’ periodontal application. Finally, the challenges and prospects of the extracellular-based therapy have been discussed.

## 1 Introduction

As a complex, chronic and inflammatory disease, periodontitis causes progressive loss of attachment, resorption of bone, and sometimes even loss of teeth (1). Epidemiological surveys have shown that 1.1 billion of severe periodontitis were detected globally in 2019 (2). Furthermore, recent data have indicated a strong association between periodontitis and several systemic conditions (3, 4), such as diabetes (5), colitis (6), cardiovascular disorders (7), rheumatoid arthritis (8), Alzheimer’s disease (9), and even pregnancy difficulties (10). Periodontitis patients require efficient treatments for better recovery and tissue regeneration (11).

Traditional periodontal treatments always focus on eliminating gingival plaque, controlling disease progression and restoring periodontal defects, through a sequence of operations involving tooth scaling, root planning, reconstructive surgery and regenerative surgery (12). However, these usual methods have limited efficacy and low predictability in clinical grounds. Achieving complete restoration of periodontium’s normal structure, function, and consistency remains a major challenge (13).

Extracellular vehicles (EVs) are natural particles secreted by all cells (14). Derived from distinct cells and biological fluids, EVs take active roles in intercellular communicating, material transferring, and even tissue regulating (15). In recent years, evidence has shown that EVs could be used to promote inflammation recovery and to facilitate tissue regeneration (16). And EVs are becoming a promising approach to improving periodontal treatment nowadays (17).

This article is the first extensive review aimed at summarizing all workflow of EVs in periodontal treatment with a broad objective. This review focuses on EV-based strategies for treating periodontitis and promoting periodontal regeneration. We begin with providing historical insights into periodontal regeneration, highlighting how evolving treatment philosophies are consistent with these concepts. After that, we discuss EVs functional mechanisms. Then, we explore the strategies of EV-based techniques in this field, generally summarize the knowledge and critically analyze the details in the utilization. Finally, we discuss the real challenges of EVs on biological mechanisms, engineering strategies, clinical translation and industrialization and the provide new perspectives for the near future.

## 2 Periodontium and periodontal regeneration

### 2.1 Periodontium

**Figure 1:**
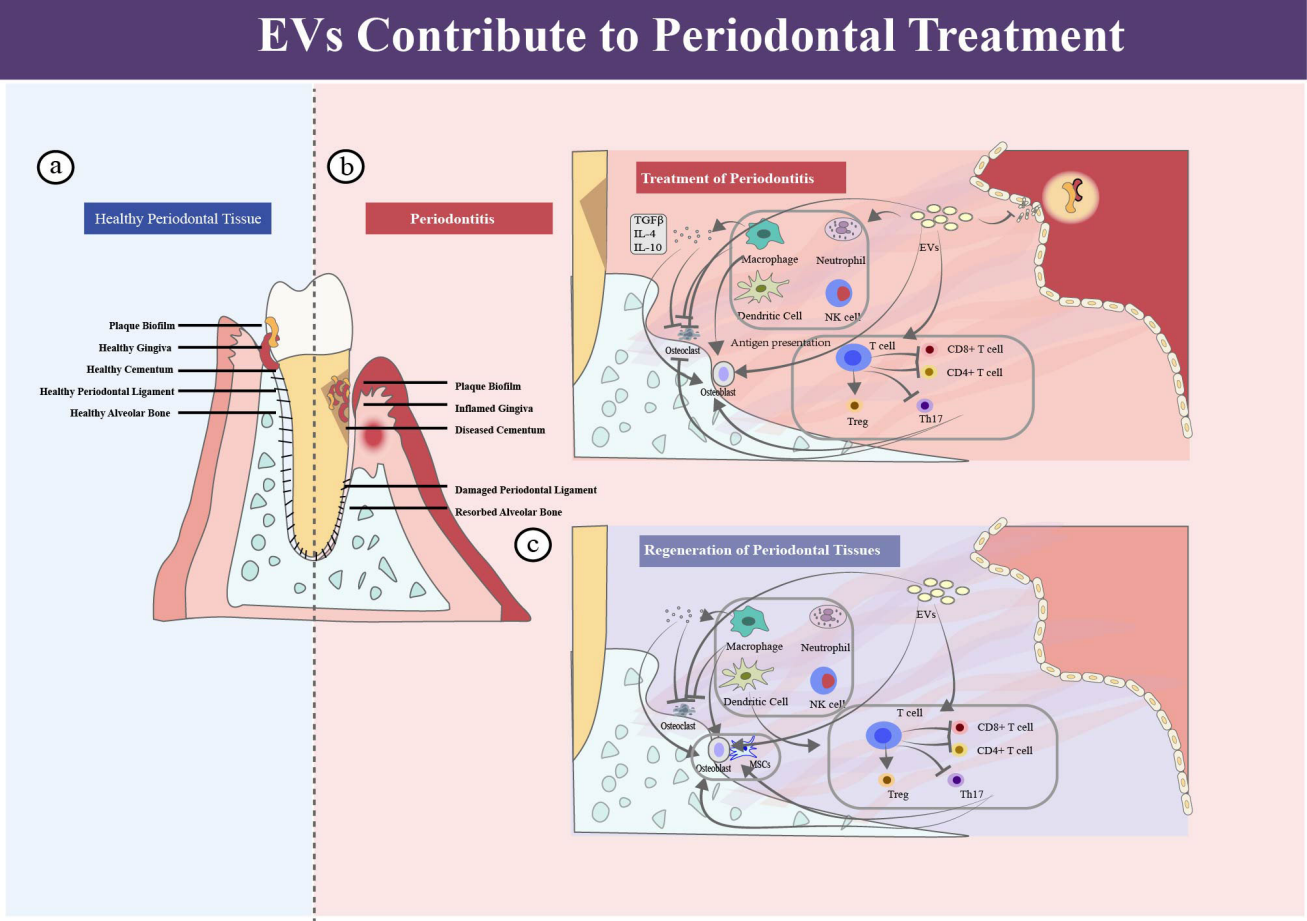
Periodontium, periodontitis and periodontal regeneration. **(a)** In healthy periodontal tissue, normal plaque biofilm exists in the gingival sulcus, and the four structures of gingiva, periodontal ligament, cementum, and alveolar bone present a healthy state. **(b)** Inflammation in periodontitis, and EVs contribute to periodontitis treatment. **(c)** EVs contribute to periodontal regeneration.

The certain term, periodontium, is used to describe the delicate structure that provides necessary support to maintain teeth in function and to protect them in health. As a hierarchical system, it consists of four principal components: gingiva, periodontal ligament, cementum, and alveolar bone (18). Covering root surface and alveolar bone in the outermost layer, gingiva, composed of epithelial and connective tissue, protects the other tissue components. Alveolar bone, the mineralized and porous tissue, supports the teeth structurally and functionally. Cementum is a calcified, avascular mesenchymal tissue that forms the outer covering of the anatomic root. And as to periodontal ligament, which continuous with the connective tissue of the gingiva and connects to the inner wall of the alveolar bone, is composed of a complex vascular and highly cellular connective tissue, and takes physical, formative and remodeling, nutritional, and sensory functions for the entire system. Although several diversities at the histological level, the four components integrate and function together as a single unit (19) (**Figure 1**).

### 2.2 History of periodontal regeneration

**Figure 2:**
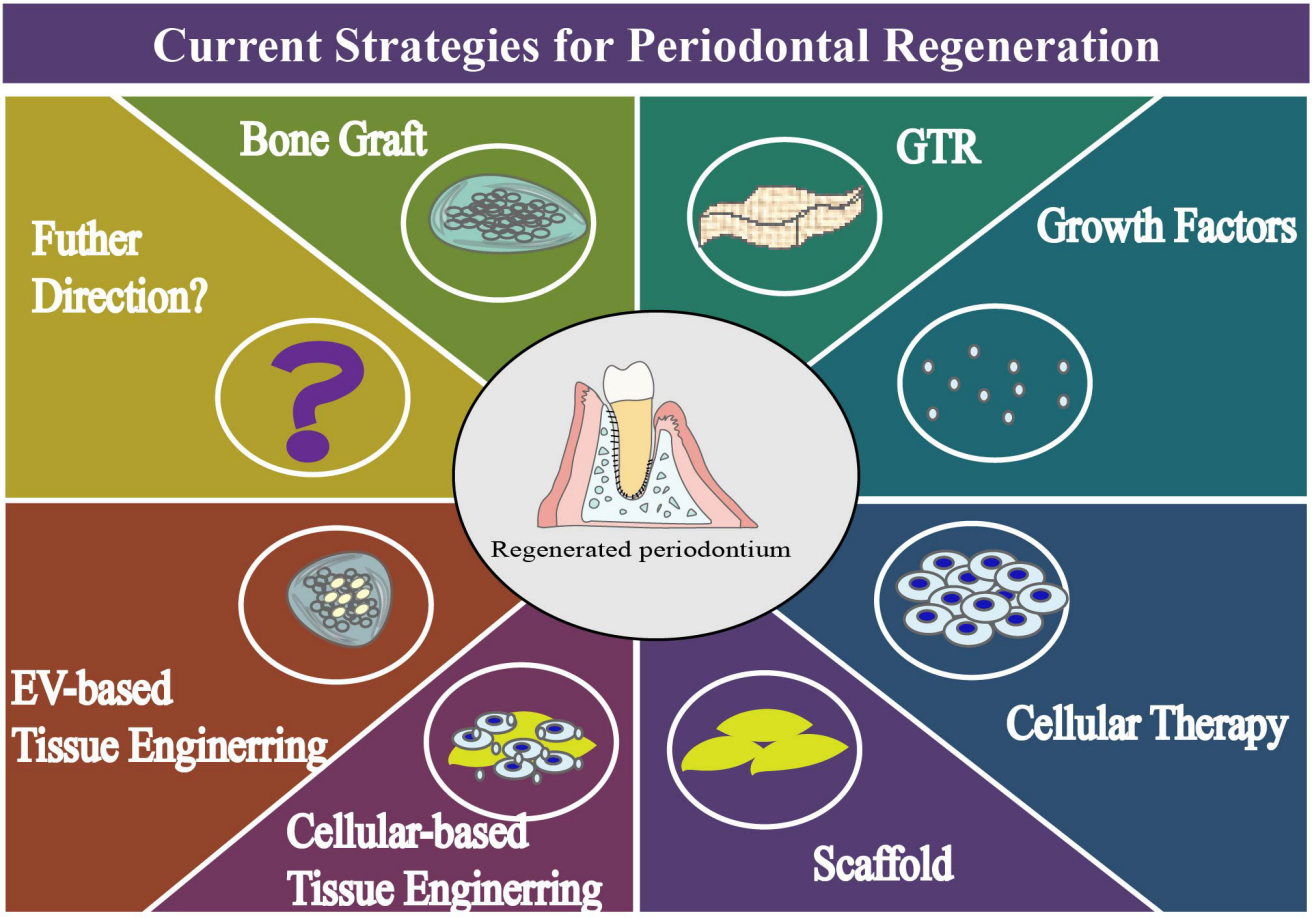
Diagram of current periodontal regeneration technology.

The restoration and regeneration of periodontal tissue have always been the goal of periodontal treatment. In history of the periodontal regeneration, scientists have made considerable efforts on a distinct range of subjects (**Figure 2**, **Table 1**).

**Table 1:** Comparison of different strategies for periodontal regeneration.

Type of periodontal regeneration technique	Type of research	Advantage	Disadvantage
GTR	Experimental study Clinical study	New connective tissue attachment Greater clinical attachment gain Greater probing depth reduction	Unpredictable Restricted indications selection; Not true regeneration of periodontium: Minor new cementum Minor bone formation, regeneration of periodontal ligament not always shown good results
Cell Therapy	Experimental study	Living cells with regenerative potential Reduce or eliminate the donor site morbidity associated with autogenous bone grafting	Unpredictable Safty consern Ethics concern Immune rejection Cost: economic, time, technical; Restricted regeneration;
Tissue Engineering	Experimental study	Economic potential Easier More time-efficient procedures.	Unpredictable Safty consern Ethics concern Immune rejection Cost: economic, time, technical; Restricted regeneration;
EV-based Therapy	Experimental study	Safety Higher transport property Suitability for storage and transport; Easily optimized Easy to use.	Unpredictable Restricted regeneration, especially the pathological tissue, such as overscale defect, Aging and systemic diseases

GTR, Guided tissue regeneration.

**Development of guided tissue regeneration.** We can read of things that happened 100 years ago, when people began their epic journey to periodontal regeneration (20). Before 1980s, long-term animal and clinical experience of periodontal tissue reconstruction has been accumulated through the application of bone graft materials, such as autogenous bone and allogeneic bone. At that time, Melcher discussed the healing potential of periodontal tissue and speculated on the interrelationship of four connective tissue cells in wound healing (21). Until 1980, with extraordinary significance, an array of experiments had been made by Neiman et al (22–24). In these original reports, periodontium was reacquired from defects of periodontitis, and a pioneering interpretation of the biological basis of regeneration was made from the experimental evidence, which was name guided tissue regeneration (GTR) (25). By placing a barrier which prevents epithelial cells from migrating along root surfaces, GTR enables the selective repopulation of connective tissue, leading to a positive outcome, such as reduced probing depth, increased clinical attachment and enhanced bone formation (26). After decades of clinical and scientific research, this procedure has been developed and validated, and has become a safe way to operate and a standard procedure of regenerate (27, 28). However, it’s the arguable outcome, limited predictability and restricted applied conditions hinder the potential of this technique.

**Growth Factors, Gene Therapy, Cell Therapy and Tissue Engineering Strategy.** 1980s is also a time to investigate the role of growth factors in promoting regeneration. Exploration of platelet-derived growth factor (PDGF) (29), insulin-like growth factors (30), enamel matrix derivative (31) in their efficacy, safety and controlled release method had been conducted in libraries. In 1993, Robert Langer and Joseph proposed the concept of tissue engineering and explained the biological and engineering principles of seed cells, scaffold materials and growth factors (32, 33). And in 2000, the gene delivery of growth factor was used for to root lining cells, and gene therapy for periodontal tissue engineering was studied at that time (34, 35). Then, the important discovery in 2004 was the extraction, characterization and culture of PDLSCs successfully (36). In the same year, BMSCs were used as auto-transplantation to promote periodontal regeneration in beagle dogs, which represented the cell therapy began its road in periodontology (37). It is expected that the implanted stem cells, with renewable potential, would promote regeneration, by directly and indirectly forming new bone tissue, periodontal membrane and cementum, through proliferation, differentiation and the release of bioactive molecules. In 2015, 3D-printed bioresorbable patient-specific polymer scaffold was investigated for periodontal reconstruction for the first time (38). The steady progress of culture technology, the gradual development of biological materials, and the practical applications of tissue engineering have resulted in endless explorations and exciting discoveries. Compared with other regenerative techniques, cell-therapy and tissue engineering merit further consideration because of the high investment of economy, the extra cost of time and the low efficiency of regeneration. Consequently, more rigorously designed studies are required in the regenerative field (39).

**EV-based therapy and endogenous tissue engineering.** Endogenous tissue engineering improves by leaps and bounds in recent decades opening up new opportunities to meet regenerative needs (11, 40). In 2019, mesenchymal stem cell exosomes were applied to promote periodontal ligament cell functions for tissue regeneration (41), and the functionally engineered extracellular vesicle were explored in 2020 (42). And the progress of the endogenous cells demonstrates the enormous potential of engineered tissue regeneration therapy.

## 3 Extracellular vesicle: a novel but promising approach to periodontal treatment

### 3.1 Overview of extracellular vesicle

**Figure 3:**
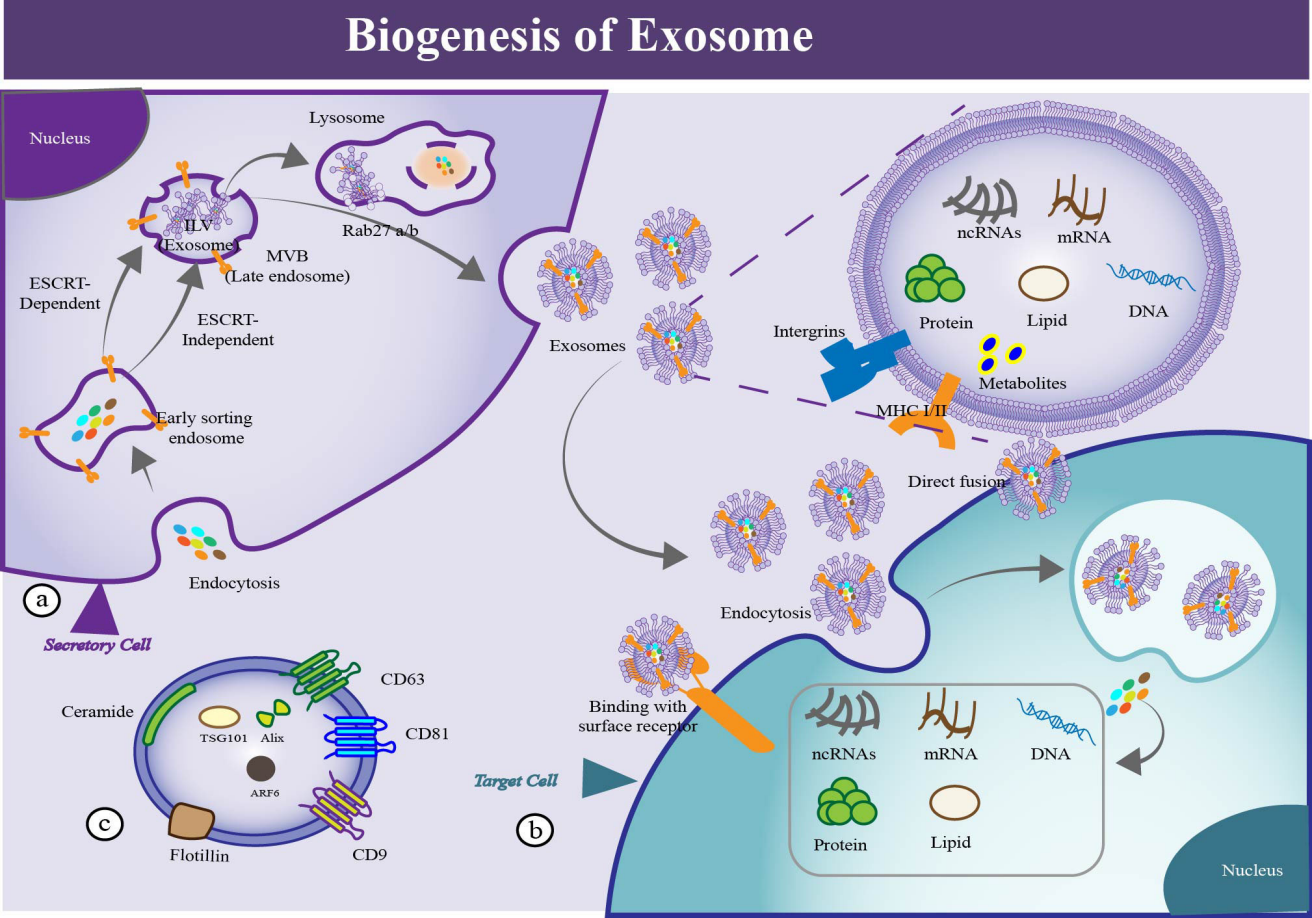
The process of EV biogenesis, uptake and identification. **(a)** Biogenesis of EVs. EVs generate from the sequential invagination of the plasma membrane, leading to the formation of intracellular multivesicular bodies (MVBs) containing intraluminal vesicles (ILVs), and are released ultimately through MVB fusion to the plasma membrane and exocytosis. **(b)** Cellular uptake of EVs. Uptake of EVs by recipient cell via multiple mechanisms, such as macropinocytosis, phagocytosis, endocytosis, membrane fusion, gap junction-mediated transfer, exchange of cargo and receptor mediated signal transduction. By these means, protein, DNA, mRNA, ncRNA, metabolites and other molecules could be transported to the recipient cell. **(c)** Identification of EVs. Several proteins could be used as markers for EVs (CD9, CD81, CD63, flotillin, TSG101, ceramide, and Alix).

Extracellular vesicles (EVs), particles with lipid bilayer, are released from cells and cannot replicate on their own. The umbrella term is recommended in the minimal information for studies of extracellular vesicles guideline in 2018 (43), which includes the several identified subtypes, such as exosomes, ectosomes, microvesicles, membrane vesicles and apoptotic bodies. Although the term “exosome” is largely used in articles, the limitation in isolation technique mixed the “exosome complex” and the true “exosomes” which derived from the endomembrane systems. To promote standardization without confusing readers, the name of EV is used in this review as the suggestion in the guideline, while the names used by the citation authors are retained when the literature is cited directly or indirectly (**Figure 3**).

### 3.2 EVs' structures, components and functions

EVs are so tiny that they are difficult to be captured with human eyes or optical microscopes. Released from both prokaryotic and eukaryotic cells, EVs play an important role not only in maintaining structural stability but also in communicating among cells. They enjoy bilayer membrane lipids, comprising sphingolipids, phospholipids, phosphatidylinositol and mono-sialo-tetra-hexosylganglioside (14, 44). The extracellular domain of the membrane contains various adhesion molecules, that promote to connect with neighbor cells. In addition, EVs also contain proteins, both as surface structures and as cargo components. Proteomic analyses of EVs demonstrated that some of these proteins are general, but some are heterogeneous, ubiquitous and cell specific. For example, integrins are discovered on the surface of EVs that exert function on fusion with other cells. Cytoskeletal proteins, annexins, GTPases, tetraspanins, heat shock proteins, apoptosis proteins, and adhesion molecules contribute to synthesis, dynamics, sort and payload. Proteins can serve as indicators for their specificity in clinical diagnostics. Some proteins could also be used to isolate EVs subpopulations and characterize them for different proteins properties. Moreover, nucleic acids, including DNA, mRNA, noncoding RNA species, can affect the behavior and phenotype of receptor cells (14). But the precious mechanisms and available features of these molecules remain unknown.

EVs produce specific effects on receptor cells through multifarious mechanisms, such as entry of intact EV by clathrincoated pit, lipid raft, phagocytosis, caveola, micropinocytosis and receptor-mediated endocytosis, release of EV contents and signaling by direct fusion and direct binding (45). By these means, the therapeutic cargo or instantaneous signal are transferred to other cells, thereby delivering drug payload or triggering cellular responses. Literature reports highlighted that EVs could not only remove excess cellular components to maintain homeostasis, but also facilitate biological processes in cell, tissue and organ levels (46). They possess valuable properties, such as anti-inflammation (47), microbiome-modulation (48), immunomodulation (49) and tissue-regeneration, making them promising candidates for treating diseases (50) (**Figure 2**). Furthermore, participating actively in cell-cell communication, signal transduction and material transport, EVs perform a variety of functions in physiological, pathological and therapeutic circumstances (51, 52).

### 3.3 Advantages and disadvantages of EVs

#### 3.3.1 Comparison between EVs and cell therapy in periodontal regeneration

The analyses illustrate that EVs are similar to their parent cells in some cases, and in several scenarios, they might be far more potent (53). Compared with cell therapy (**Table 1**), EVs gain advantages of: (1) Enhanced safety. They are safer than living cells with low immunogenicity. They produce fewer side effects and fewer possibility of tumor formation (54). (2) Superior transport capability. Their bilayer architecture endows them higher integrated ability into target cells. Also, they can be readily manipulated and genetically engineered, which provides excellent pharmacokinetic properties and tissue-targeting capacities. Released in a nano-size form, they are able to travel across capillaries and many small barriers freely, especially blood–brain barriers (55). (3) Easier to storage and transport. They can be kept for a long time without losing their characteristics. (4) Optimizable efficacy. Although the content of EVs is like that of their source cells, optimizing EVs in composition and characteristic can significantly improve their treatment efficiency and consequence. (5) Easier application. EVs not only could be directly injected, but also could combine with scaffold materials. Cell therapy can also enter the body by these means, but the condition requirements of living cells are much higher than that of the non-living EVs.

Disadvantages of EVs: A key disadvantage of EV-based application lies in their limited regeneration ability. For example, the regenerative performance of endogenous cells is weakened by the harmful effects of local and systemic factors, especially in inflammatory environments and aging individuals. Consequently, EVs exhibit reduced therapeutic efficacy. For further details on the application of EVs, please refer to the relevant review (52, 53) and see **Table 1** for additional information.

#### 3.3.2 Comparison between EVs and liposome in drug loading

Compared with the conventional carrier, liposomes, EVs do have some advantages (44, 56, 57). (1) EVs are safer than artificial liposomes. EVs are derived from natural cells with cellular lipids and negligible toxicity. (2) They have superior transport capability. They could travel across blood-brain barriers. (3) They could be manipulated in genetical level. EVs can be engineered in both active and passive approaches, while liposomes could only be regulated by the passive route, which provide additional possibilities for the source of content drugs encapsulated by EVs.

There are some drawbacks of EVs in drug loading. (1) EVs loading efficiency is limited by their intrinsically components. The cargoes with the natural EVs possess the highly heterogeneity and variability, which make the loading performance of EVs unstable to product and difficult to predict. In addition, this will also create obstacles for loading of multifunctional cargoes. (2) A lower degree of industrialization. Lower yield, high time- cost and effort-consuming restrain the application of EVs at the present time. (3) Poor reproducibility. Heterogeneity of EVs, non-standardized manufacturing process and numerous influencing factors make it difficult for EV derivatives to maintain consistency. (4) Unspecific targeting. Natural EVs lack the specific targeting and tissue-homing capacity. (5) Exogenous packaging technology may cause damage to the loaded drug.

### 3.4 New achievements of EVs

EVs’ use in scientific research and clinical application has made good progress due to their inherent properties as natural EVs, engineered capabilities as therapeutic vehicles and integrated cargoes as delivery systems (53). Over 50000 publication could be searched on EV’s topic and the trend continues dramatically. Currently, EVs have gained remarkable achievements: Engineered EVs treatment in Acute Liver Failure (58), EVs facilitate diabetic wound healing (59), EVs alleviate proinflammatory cascades in Alzheimer’s disease (60) and EVs’ impressive potential in anti-tumor immunity (61).

## 4 EVs perform valuable functions in periodontal treatment

**Figure 4:**
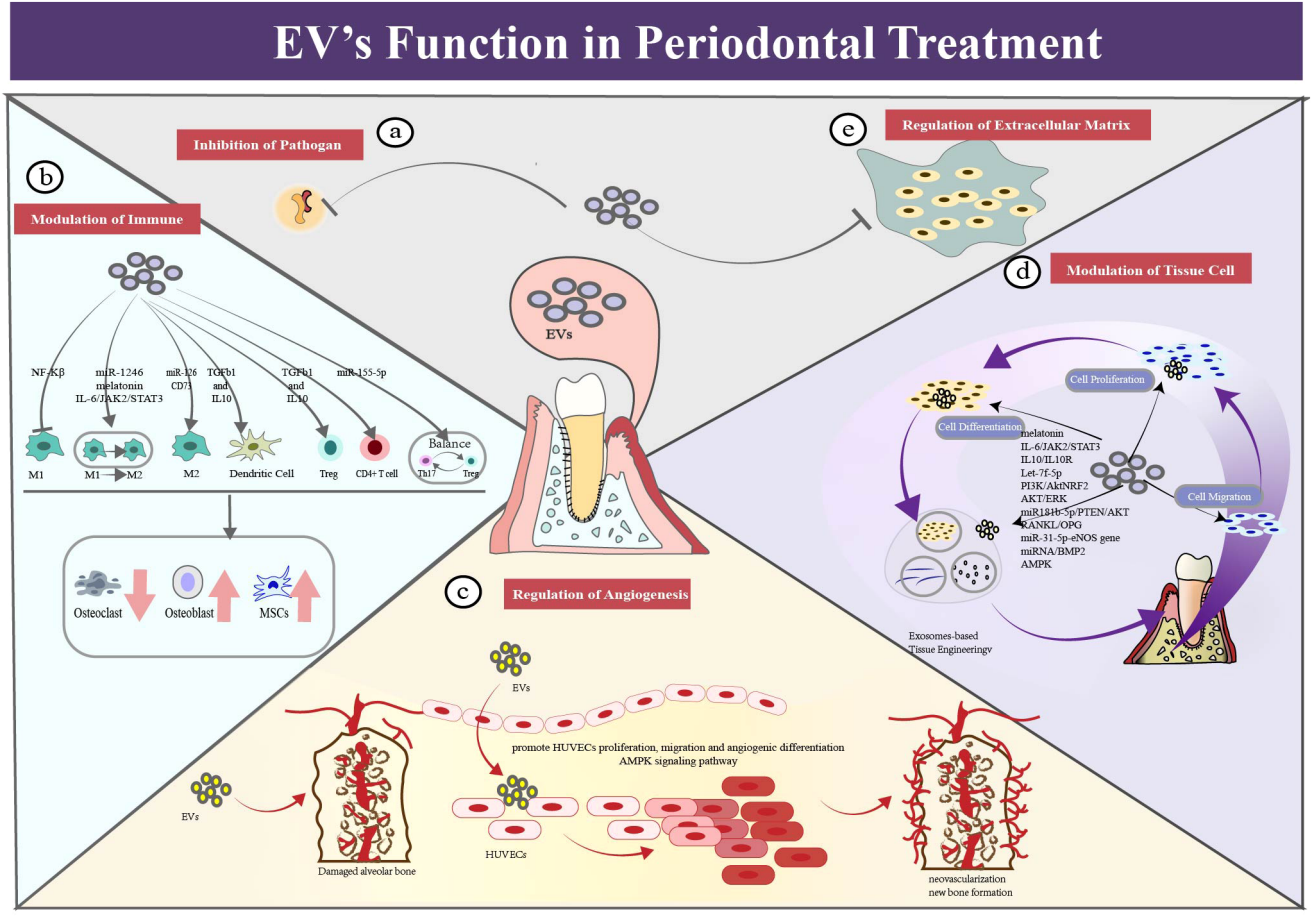
EV’s functions in periodontal therapy. **(a)** EVs exert a function of assisting in antimicrobial regulation. Exosome-like nanoparticles could serve as a potential therapeutic strategy for periodontitis. **(b)** EVs exert a function of immune modulation in periodontal therapy. EVs could regulate different phenotypes of macrophages to the anti-inflammation state. It can also regulate dendritic cells by cytokines (TGF-b and IL-10) delivery which alleviate bone resorption. EVs could function in regulating T cells, such as CD4+ T cell, Treg cells and Th17 cells. **(c)** EVs exert a function of regulating angiogenesis in periodontal therapy. EVs could promote migration, proliferation and differentiation of f human umbilical vein endothelial cells((HUVECs), which contributing the repair and regeneration of periodontium. **(d)** EVs exert a function of modulating activity of tissue cells in periodontal therapy. EVs could promote migration, proliferation and differentiation of tissue cells, which contributing the regeneration of periodontium. **(e)** EVs exert a function of modulating the extracellular matrix network, especially in the periodontal field.

EVs fulfill multiple functions in the periodontal field. Such as, EVs could inhibit inflammatory responses locally. They could regulate the remodeling of damaged tissue, such as cementum, periodontal ligament, alveolar bone tissue, extracellular matrix and vascularization. It is worth noting that EVs have an extensive impact on the periodontal regeneration (**Figure 4**).

### 4.1 EVs assist in antimicrobial regulation

EVs assist the regulation of local pathogens (62). For example, ginger extracellular vesicle-like nanoparticles (GELNs) were specifically absorbed by the periodontal pathogen, *Porphyromonas gingivalis. (P. gingivalis)* GELNs significantly reduced the harmful effects of *P. gingivalis* by binding to its heme-binding protein 35 on the bacterial surface and interacting with GELN cargo molecules such as phosphatidic acid and miRNA. This result demonstrated that the exosome-like nanoparticles could serve as a potential therapeutic strategy to prevent or treat chronic periodontitis in mouse models (63, 64). In the available literature, no evidence of using natural EVs directly for antibacterial application has been found.

### 4.2 EVs regulate immune inflammatory response

EVs can modulate macrophage polarization in local periodontal tissue, aiding in periodontal immune regulation (65–68). From healthy state to periodontitis condition, macrophages play distinct roles through phenotypic transformation. The study of Ru Wang (69) supported this notion by co-incubation of gingival marrow stem cell (GMSC)-derived exosomes with macrophages. The experiment showed significantly decreased level of M1 markers, decreased number of pro-inflammatory cells and reduced destruction of bone models. In addition, EVs have performed functions in other immune cells. This is supported by evidence from the Zhang Yong group. After routine injections of marrow stem cell(MSC)-exosomes, the periodontitis mice exhibited lower bone loss, better bone formation and fewer inflammatory cells compared with the control group (70), which indicated that exosomes could alleviate periodontitis by mediating of differentiation CD4+ T cells and relieving imbalance of Th17/Treg cells. Ya Zheng further discovered that periodontitis patients exhibited a Th17/Treg imbalance in their peripheral blood, with upregulation of Th17 cells or downregulation of Treg cells. They also found that the PDLSCs exosomes mitigated the inflammatory microenvironment via the regulatory network of Th17/Treg/miR-155-5p/SIRT1 (71). In addition, exosomes inhibited the maturation of dendric cells and the induction of Th17 effectors, but promoted the recruitment of T-regulatory cells, leading to the decline in resorptive cytokines and osteoclastic loss (71).

### 4.3 Modulation of periodontal tissue cells through EVs

**EVs contribute to cementum regeneration**. Cementum, a mineralized tissue covering tooth root surfaces, is a vital component of the periodontal tissue. Despite its importance, the specific mechanisms and effective strategies for cementum regeneration remain unclear. Previous studies have investigated the applications of growth factors or enamel matrix derivatives. Here, we present the evidence which supports EVs as a promising delivery vehicle to enhance the regenerative outcome (72–75). For example, Yi Zhao’s animal experiment demonstrated that exosomes from M2-polarized macrophage led to enhanced cementoblast mineralization (76). Additionally, a study of Shengnan Li et al. found that exosomes derived from human periodontal ligament stem cells (PDLSCs) promoted cementoblast activity via the PI3K/AKT signaling pathway, enhancing the migration, proliferation, and mineralization of OCCM-30 cells (77).

**EVs regulate formation of periodontal ligament** (67, 78–82). For example, human MSC-derived exosomes have been used to treat periodontal intrabony defects by local administration. Loaded onto a collagen sponge, rats in the exosome-treated group exhibited enhanced efficiency in repairing periodontal defects, with new bone and new periodontal membranes forming, accompanied by increased cell infiltration and promoted cell proliferation (72).

**EVs could be used to regulate alveolar bone formation and absorption** (41, 42, 50, 67, 75, 77, 81–92). Relevant theories can be referred to the review literature (93–95). EVs are expected to inhibit bone resorption and promote bone formation. Preclinical evidence has demonstrated that oral MSC-derived EVs could promote osteogenic repair in bone formation and periodontal defects (96–98). For example, MSC exosomes enhanced the migration and proliferation of periodontal ligament cells by activation of CD73-mediated adenosine receptor and stimulation of the pro-survival AKT and ERK signaling pathways (41). Combined with Matrigel or β-TCP, exosomes derived from h-PDLSCs enhanced bone formation in alveolar defects. That showed exosomes’ potential to restore the bone-forming ability of stem cells in the pro-inflammatory setting (50).

### 4.4 Modulation of extracellular matrix via EVs

EVs can modulate the extracellular matrix (ECM) network, especially in the periodontal field. ECM is a non-cellular three-dimensional structure, providing structural integrity and cellular regulation. As a highly dynamic network, ECM is essential to tissue maintenance, such as synthesis of substances and modification of chemicals (99). Deregulation of ECM structure is linked to diverse pathological conditions, such as osteoarthritis and periodontitis. It is also essential for tissue regeneration, that ECM could be tightly regulated instead. For example, derived from MSCs, functionally engineered EVs interacted with ECM proteins and peptides, leading to a fourfold increase in bone regeneration, compared with the control group in calvaria defect models (100).

### 4.5 Regulation of angiogenesis via EVs

EVs are considered as a novel approach to regulate angiogenesis (101). Blood vessels and lymphatic vessels serve their functions in resolving inflammation and promoting tissue regeneration. Beneath the gingival sulcular and junctional epithelium of periodontitis individuals, vascular networks undergo significant changes in microcirculation and vascular development. Alongside veins, nerves, and lymphatics, blood vessels carrying seed cells, growth factors and nutrients enter the interdental septa during periodontal regeneration process. For example, pre-treated with hypoxia, dental pulp stem cell-derived exosomes (DPSC-Exos) improved the proliferation, migration, and formation of human umbilical vein endothelial cells (HUVECs) and partially modified their proteome profile (42). Additionally, histological and immunohistochemical analysis showed that DPSC-Exos stimulated formation of new blood vessels in wound healing. In the vitro study, it was demonstrated that DPSC-Exos enhanced the ability of HUVECs to migrate, proliferate and regeneration through the Cdc42/p38 MAPK pathway (102). A comprehensive review has been published by Yunhao Qin (103).

## 5 EV-based strategies for periodontal treatment

**Figure 5:**
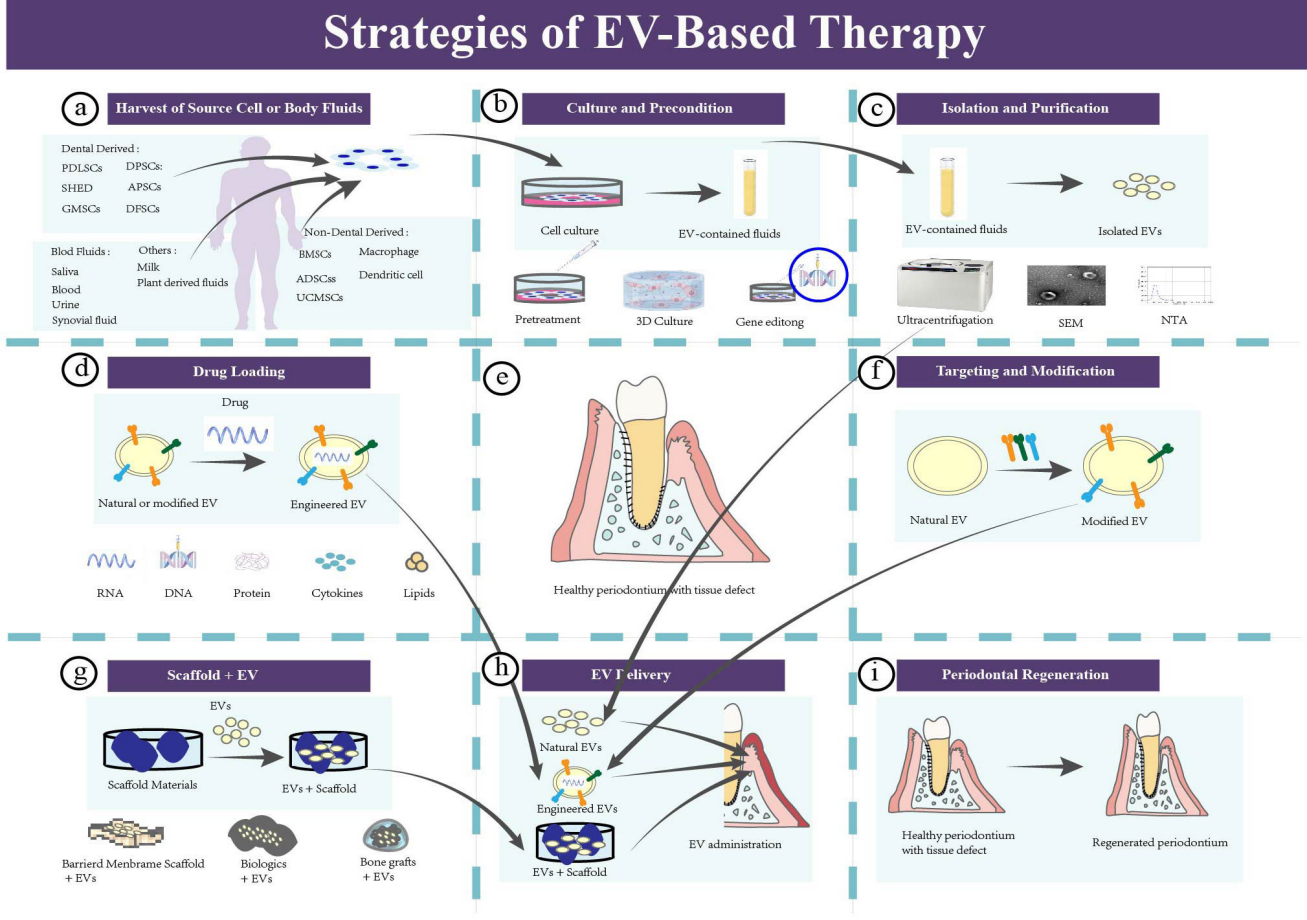
The strategies of EV-based therapy in periodontal regeneration. **(a)** Harvest of source cells or body fluids for isolating EVs. **(b)** Culture and pretreating the parent cells. Natural cells should be cultured for EVs insolation, and pretreating those parent cell could enhance the intrinsic properties of EVs. Common pretreatment methods include mechanical and physical pretreatment, chemical pretreatment, biological pretreatment, 3D culture, and gene editing. **(c)** Isolation, purification, and identification of EVs. **(d)** Drug loading of EVs. **(e)** Healthy periodontium with tissue defect. **(f)** Targeting and modification of EVs. **(g)** EVs-based tissue engineering strategy. **(h)** EVs delivery. Administration of EVs to the periodontal tissue defect. **(i)** Periodontal regeneration.

In this section, an overview EV-based strategies for periodontal treatment is provided. It is subdivided into seven parts according to the general workflow (104) (**Figure 5**) (**Table 2**).

**Table 2:** Workflow of EV-based strategy.

Steps	Reporting Data
**Harvest of Samples**	Source of materials
	Collection protocols
	Storage parameters
**Culture and Precondition**	Cell culture parameters
**EV Separation**	Separation protocols
**EV Characterization**	Characterization protocol
	Quantification
	Characterization
	Localization
	Safety
	Purity
**EV Loading/Modification**	Protocol
	Endogenous loading/modification
	Exogenous loading/modification
	Cargo information
**EV Assessment**	Assessment protocol
	Parameters: efficiency, capacity
**EV + Scaffold**	Biomaterials
	Synthetic material
**EV Administration**	Local injections
	Systematic administration
	Delivery with biomaterials
**EV Functional Effects**	Experiment protocol
	Appropriate negative EV controls
	Dose-response and time-course studies(physiologically informed)
	Assess the effects of pre-analysis factors

### 5.1 Sample harvesting

The initial step is sample harvesting for EV isolation. Rigorous preparation, based on complex demands and distinct sources, is crucial for designers to achieve optimal results and regeneration goals. At present, there is no international consensus on the standard scheme for EVs’ source selection, and researchers must refer to existing literature which has provided a series of clues for the consideration on this topic (17). Therefore, we have summarized the following lists to help think about the problem.

Source samples should provide appropriate characteristics for EVs:

1. Valuable functions
2. Tissue-homing property
3. Low immunogenic, low toxicity, no oncogenic considerations
4. Genetic stability, no host-cell impurities (pathogens, especially viruses)
5. Healthy donors

**Source samples should provide a scalable and sustainable approach for EV harvesting:**

1. Quality of EVs: stable, sustainable
2. Quantity of EVs: scalable
3. Manufacturing technology: practical, easy to harvest, to manipulate and to maintain
4. Cost effective

**The selection of sample sources should avoid**:

1. Highly invasive harvesting procedures
2. Highly challenging work
3. Unlikely to reach clinical implementation
4. Low-cost performance
5. Samples with pathological status and infection risk

To the first, source samples are required to provide EVs with appropriate characteristics (57, 105). The properties of EVs are firstly derived from inherent potential of their parental cells, such as tissue regulation capability, homing/targeting properties, immunogenic characteristic, genetic stability and anti-inflammatory potential (51). In the field of periodontal regeneration, it is common to use mesenchymal stem cells, for their self-renewal potential and multi-directional differentiation ability. Common stem cells include bone marrow stem cells (BMSCs) (106), adipose-derived stem cells (ADSCs) (107), and other mesenchymal stem cells, like umbilical cord stem cells (101). Also, oral tissue regeneration relies on various odontogenic stem cells, such as PDLSCs (108), DPSCs (102), dental follicle cells (109), stem cells from human exfoliated deciduous teeth (SHED) (110), GMSCs (109) and stem cells from the apical papilla (111). In addition, some immune cells have been studied for inflammation regulation and disease treatment, such as macrophages (78) and dendritic cells (112). Still another, plant cells, involving ginger (113) and onion (114), have been tested for more innovative and convenient options. For example, for enhancing periodontium formation, researchers could select PDLSCs, whose EVs have been indicated the properties of inhibiting osteoclast differentiation, reversing cell aging, promoting cell proliferation and facilizing inflammation recovery (50, 115).

Secondly, source samples should provide scalable and sustainable EVs. The harvesting of sample sources and the manufacturing of EV products must be scalable and repeatable, so that the quality, quantity, cost and efficacy could meet the needs of actual production and clinical application. For example, when engineering modification is required, specialized cell lines like 293T/17, known for their genetic editing capability, can be commonly applied (67). Then, blood, urine, saliva and milk, body fluids and solid tissue provide researchers with convenient sources for EVs.

Thirdly, researchers should avoid the selection of highly invasive samples, challenging manufacturing procedures, low cost-effective products, research projects with difficulty in clinical translation and parental tissue with risks in contamination (57). Genetic variability, epigenetic modification, environmental changes and pathological states could influence the functions of EVs. For example, exosomes from healthy periodontal ligament stem cells led to enhanced mineralized nodule formation and increased osteogenic indicators in the inflamed PDLSCs. Mechanistically, these exosomes inhibited the over-activation of the canonical Wnt pathway, thereby restoring the osteogenic potential of the inflamed PDLSCs (50).

### 5.2 Cell culture and pretreatment

Another key step is to culture and pretreat the obtained samples. Once the sample is collected, researchers need to culture, preprocess and store it for subsequent isolation and purification. In the complex process, the methodologic details should be recorded carefully. With guideline of Minimal Information for Studies of Extracellular Vesicles (MISEV) (116), a common basis for standardization had been set, which is crucial for quality control and outcome comparison. For example, when parent cells have been collected, characteristics of the cells and identification of cell lineage must be fully recorded. In addition, their medium components, culture conditions, stimulation and other treatments should be described in detail. Besides, if there are contaminations or infections, the specific situation and treatment operation should be carefully reported. Furthermore, the presence of apoptotic and dead cells greatly affects the characteristics of vesicles. Therefore, the percentage of these cells should also be faithfully recorded. Then, sometimes, natural EVs have been used in periodontal treatment (**Tables 3**, **4**). For example, Shen, Z. extracted exosomes from DPSCs. Incorporated into chitosan hydrogel, these exosomes facilitated the transition of macrophages from a pro-inflammatory to an anti-inflammatory phenotype, and accelerated healing of the alveolar bone and periodontal epithelium in periodontitis-afflicted mice (66).

**Table 3:** EV-based therapeutic applications in periodontitis (Natural EVs).

EV source	EV-cargo	Research model	Outcome		Ref
			*In vitro*	*In vivo*	
DPSC-EXO		Rat ligature periodontitis model	PDLSCs:Promoted proliferation, Promoted migration, Promoted osteogenesis Mø: Promoted M1 to M2	Alveolar bone:Reduced resorption; Periodontal epithelium:promoted healing	(67)
PDLSC-Exos	MicroRNA-141-3p	*In vitro*: High glucose-induced senescent model	Senescent PDLSCs: Promoted rejuvenation	Alveolar bone:Promoted healing;	(115)
DPSC-Exo	MiR-1246	Mice-periodontitis model	Mø: Promoted M1 to M2	Periodontal epithelium:Promoted healing; Mø:Promoted M1 to M2	(66)
GMSC-Exo			PDLSCs:Promoted osteogenesis		(109)

DPSC-EXO, Dental pulp stem cell-derived exosomes; PDLSC-Exos, human periodontal ligament stem cell-derived exosomes; DPSC-Exo, dental pulp stem cell-derived exosomes; GMSC-Exo, Human gingival mesenchymal stem cell-derived exosomes; PDLSCs, Periodontal ligament stem cells; Mø, macrophages; M1, Classical activated macrophages; M2, Alternatively activated macrophages.

**Table 4:** EV-based therapeutic applications in periodontal regeneration (Natural EVs).

EV source	EV-cargo	Research model	Function		Ref
			*In vitro*	*In vivo*	
SHED-derived exosomes		Periodontal defect rat models	HUVECs: Promote proliferation, migration, angiogenic differentiation BMSCs: Promoted proliferation, migration, osteogenesis	Alveolar bone: Promoted defect repair New bone formation, neovascularization	(85)
M0 EVs M1 EVs M2 EVs	M1 EVs: miR-155, M2 EVs: miR-378a	Rat calvaria bone defect model	M0 and M2 EVs: Promoted repair/regeneration M1 EVs: Inhibited bone repair negatively regulated the BMP signaling pathway	M1 EVs: Impaired bone regeneration. M0 EVs: Promoted bone regeneration. M2 EVs: Promoted bone regeneration	(117)
MSC exosomes		Rat periodontal defect model	Enhanced proliferation and migration	Promoted regeneration of critical-size periodontal defects	(41)
PDLSCs-Exos		Rat Alveolar Bone Defect Model	Promoted the proliferation osteogenic differentiation	Promote repairment	(108)
Osteoblast-Derived Matrix Vesicles	miRNA		Decreased osteoblast differentiation		(83)

SHED, stem cells from human exfoliated deciduous teeth; Mø, macrophages; M1, Classical activated macrophages; M2, Alternatively activated macrophages; MSC exosomes, marrow stromal cells derived exosomes; PDLSC-Exos, human periodontal ligament stem cell-derived exosomes.

To enhance EVs properties and achieve high-performance products, pretreating parent cells is an important approach. Common pretreatment methods comprise culture regulation, biochemical stimulation and physical pretreatment (95), such as Tumor necrosis factor-α(TNF-a) (118), Lipopolysaccharide(LPS) (92), IL-4 (117), IL-10 (84), 3D culture (70), high glucose culture (119), hypoxia culture (120) and mechanical stretch (121). On the one hand, pretreating strategies preserve the original function and primary integrity. On the other hand, the produced EVs become more precise and more efficient for practical treatment (107, 122). However, the cells’ responses to the external stimuli might be variable, and the EVs’ properties are not fully defined, making it challenging to use this method accurately. For example, LPS-preconditioned dental follicle cell-derived sEV (L-D-sEV) promoted proliferation, migration, and differentiation of PDLSCs from periodontitis (p-PDLCs) in a dose-dependent and saturable manner. The L-D-sEV demonstrated a slight advantage over conventionally cultured dental follicle cell-derived sEV in enhancing p-PDLCs’ differentiation. Moreover, L-D-sEV leaded a partial reduction in the RANKL/OPG ratio and revealed advantageous in pathological models, through aiding the restoration of lost alveolar bone in the initial treatment phase and preserving alveolar bone levels in the later treatment phase (92).

In summary, standardized design of culture strategies and comprehensive documentation of practical protocols are very important for the rigor, reliability and repeatability of experimental research.

### 5.3 EVs' separation and characterization

Separation is a crucial step in the preparation of EVs. Expected to be scalable and robust, separation depends on EVs’ physiological characteristics such as size, density, charge, and surface structure, and different approaches determine the yield, specificity and purity of products. Common separation approaches consist of didderential ultra-centrifugation, density gradient centrifugation, precipitation, filter concentration, size-exclusion chromatography, immunoaffinity capture techniques, and microfluidic techniques (116). Among them, the most specific method is the immune-precipitation method, and the highest recovery rate of outer vesicles is the precipitation method. At present, the most widely used technique in literature is ultra-high-speed centrifugation. Different centrifugal forces are used to separate solutes according to their volume, mass and sedimentation coefficient. This method enjoys its advantages such as low cost, large quantity and large yield. But its low purity and heavy workload hinders the application of this technique. Researchers can improve the quality of EV through the combination of different approaches.

After the isolation procedure, researchers should characterize them to verify their existence, evaluate their quantity and identify their purity. Orthogonal methods are recommended, for no single technique is adequate for all needs. According to MISEV2024 (116), the quantitative measures and approximation estimate of EV source (e.g., number of secreting cells, volume of biofluid, mass of tissue) and the EV abundance should be made. The identification of components associated with EVs and the proportion of the non-vesicular, co-isolated components should be demonstrated. In detail, the total protein, total lipids, total RNA, EV morphology, protein composition, non-protein markers and localization of EV-associated components are required. These characteristic should be measured by Flow cytometry, PCR, Western Blot, Nanoparticle tracking analysis, Microscopy-based methods (123). Besides, the safety measurement of EV products is also in need, including their biocompatibility, immunogenicity, tumorigenicity and toxicity.

### 5.4 EVs' engineering and modification

Scientists are always exploring more stable, efficient and quantifiable strategies, especially at a genetical level. (**Tables 5**, **6**) In parent cells, genetic engineering approaches may well modify RNA expression and protein production, thereby altering the contents and surface features of their EVs and changing their function. For example, Xin Huang etc. designed the M2-like macrophage-derived exosomes by the genetic engineering technique. To obtain permanent M2-like macrophages, they silenced the key gene, casein kinase 2 interacting protein-1 (Ckip-1), which promised not only a long-term specific characteristic, but also a promoted mineralization effect. They collected the exosomes from these engineered cells and measured their therapeutic capability on periodontal regeneration. These exosomes (sh-Ckip-1-EXO) rescued the cementoblast whose function had been suppressed by *Porphyromonas gingivalis*. At a molecular level, sh-Ckip-1-EXO enhanced the cementoblast mineralization and cementogenesis by delivering the key molecule of Let-7f-5p and silencing the inhibitor of cementum formation (75). Chun-Chieh Huang modified HMSCs genetically to carry out this exploration. HMSCs, with promoted osteoinductive effect, expressed BMP2 constitutively. For this reason, they are expected to produce exosomes with BMP2 protein and osteoinductive function. Then, in the calvarial defect models, these engineered HMSC exosomes indeed enhanced the bone regeneration. However, BMP2 protein was not detected as the constituent in these functionally vesicles. Further research showed that the exosomes, which enhanced the BMP2 signaling pathway without BMP2 protein, possessed alerted miRNA after gene modification. This result demonstrated that the effect of EVs had be improved by genetic modification of their original cells (42).

**Table 5:** EV-based therapeutic applications in periodontitis (Engineered EVs).

EV source	EV-cargo	Research model	Outcome		Ref
			*In vitro*	*In vivo*	
TNF-a-preconditioned GMSC-derived exosomes		Mice ligature periodontitis model	Mø: Promoted to M2	Alveolar bone: Reduced resorption;	(73)
LPS preconditioned dental follicle cells derived small extracellular vesicles		Experimental periodontitis rats	PDLCs: Promoted migration, Promoted osteogenesis	Early stage: Repaired lost alveolar bone; Late stage: maintained level of alveolar bone	(53)
PDLSCs-Exo	MiR-31-5p	Mice ligature periodontitis model	osteoclast: Inhibited osteoclast differentiation	Alveolar bone:Reduced resorption	(75)
M2-exos	IL-10 mRNA	Murine periodontal disease model	BMDM: Promoted osteoclast differentiation; BMSCs: Promoted osteogenesis	Alveolar bone:Reduced resorption	(55)
3D-exos	MiR-1246	Ligature-induced model of periodontitis	Th17cell/Treg: Recovery of balance	Enhanced ameliorating periodontitis	(56)
Ckip-1-silenced macrophages	Let-7f-5p		Cementoblast: mineralization. Rescued cementogenesis	Rescued cementoblast mineralization and cementogenesis	(62)
293T/17 cells overexpressing the C-X-C motif chemokine receptor	MiR-126	Rat ligature periodontitis model	Mø: Promoted to M2	Mø: Promoted to M2; Inhibited osteoclast differentiation Alveolar bone: Reduced resorption	(67)
regDC EXO	TGFB1 and IL10		DCs: Induced immune regulatory CD4T-cell: inhibited proliferation; Tregs: promoted induction	Recipient DCs: Suppressed maturation Th17 effectors: Appressed induction. Treg: Promoted recruitment;Alveolar bone:Reduced resorption	(47)

TNF-a-preconditioned GMSC-derived exosomes, gingival tissue-derived MSCs preconditioned with TNF-a; PDLSCs-Exo, periodontal ligament stem cells derived exosomes; M2-exos, reparative M2-like macrophages; 3D-exos, Exosomes derived from 3D-cultured MSCs; regDC EXO, immuneregulatory; Mø, macrophages; M1, Classical activated macrophages; M2, Alternatively activated macrophages; BMSCs, Bone marrow stromal cells; BMDM, Bone marrow derived macrophage; DCs, Dendritic cells; T cells, T lymphocyte; Th17cell,T helper cell 17; Treg, Regulatory T cells; CD4T-cells, CD4 effector T cells; PDLCs, periodontal ligament cells.

**Table 6:** EV-based therapeutic applications in periodontal regeneration (Engineered EVs).

EV source	EV-cargo	Research model	Function		Ref
			*In vitro*	*In vivo*	
Functionally engineered EVs from genetically modified human bone marrow derived MSCs constitutively expressing BMP2	altered miRNA composition	Rat calvarial defect model	Enhanced osteogenic differentiation	Promoted enhanced bone regeneration	(42)

Bone morphogenetic protein-2.

### 5.5 EVs for drug loading

Drug loading provides another choice for EV-based treatment. EVs have been explored as novel carriers for small molecules, proteins and nucleic acids delivery, in the light of their biological, structural and pharmacological characteristics (125) (**Table 7**).

**Table 7:** EV-based therapeutic applications in periodontitis (Drug Load).

EV source	EV-cargo	Research model	Outcome		Ref
			*In vitro*	*In vivo*	
M2-exos	Melatonin	Rat ligature periodontitis model	Mø: M1 to M2 hPDLSCs: osteogenic and cementogenic differentiation restored the impaired function	Reversed alveolar bone loss	(78)
P2X7R gene-modified stem cell-derived exosomes	MiR-3679-5p, MiR-6515-5p, MiR-6747-5p		PDLSCs: Reduced inflammation-mediated changes		(124)

M2-exos, M2 macrophage-derived exosomes; PDLSCs, Periodontal ligament stem cells; Mø, macrophages; M1, Classical activated macrophages; M2, Alternatively activated macrophages.

Drug loading processes consist of endogenous (pre-secretory) loading strategies and exogenous (post-secretory) loading strategies (44). First, endogenous loading strategies involve modifying parental cells through gene editing and loading parental cells through co-incubation. By these means, EVs preserve their integrity when harvesting specific proteins, nucleic acids and drug molecules. Endogenous loading approaches also allow scientists for accurate prediction and stringent regulation. However, the primary limitation is the difficulty in effective loading (126). For all this, endogenous loading strategies have shown potential in application for periodontal regeneration. For example, exosomes containing miR-126 (CXCR4-miR126-Exo) were effectively created through the transient transfection of miR-126 into 293T/17 cells that overexpressed CXCR4. It increased the anti-inflammatory cytokines, decreased the bone resorption and ultimately halted the periodontitis advancement (127).

Secondly, to address the limitations of the endogenous methods, exogenous (post-secretory) loading approaches have been developed. Exogenous loading strategies incorporate proteins, nucleic acids, bioactive molecules and drugs into isolated EVs, offering simpler protocols, greater stability and continued scalability for large-scale production in efficient drug delivery systems (128). In the periodontal field, particularly applied techniques include co-incubation, electroporation, sonication, freeze–thaw cycles and extrusion (129). For example, melatonin, a crucial hormone-like substance, acts as an antioxidant in cellular physiology. It modulates tissue inflammation and regulates cell apoptosis under various pathophysiological conditions. Ya Cui and colleagues loaded melatonin into M2 macrophage-derived exosomes (Mel@M2-exos) to explore its application potential (78). Released in the periodontium, these Mel@M2-exos restored the osteogenic capacity of the inflammatory human periodontal ligament cells (hPDLSCs). Animal experiments demonstrated that Mel@M2-exos in gelatin methacryloyl hydrogels significantly accelerated periodontal bone in rats with ligation-induced periodontitis (78). Mahmoud Elashiry loaded dendritic-cell exosomes with TGF-B1 and IL-10 (112). Then, to explore their therapeutic effect, these exosomes were administrated locally and intravenously. Immunoregulatory DC exosomes (regDC EXO) were effectively taken up by DCs and T cells, protecting TGF-B1 and IL-10 and delivering them at the same time. As a result, the local alveolar bone resorption was inhibited, which illustrated that the exosomes, loaded with effective cytokines, could provide a promising outcome for periodontitis treatment.

The drug loading performance of EV needs to be evaluated by multiple indicators. Common parameters include safety, efficacy, biocompatibility, stability, pharmacokinetic parameters, pharmacodynamic parameters and loading parameters. It is very important to detect these indicators, because they can provide researchers with specific references in dosage, concentration and strategy design. For example, the encapsulation efficiency, parameter for exogenous loading, refers to ration of loaded fraction compared to the total loaded amount (104). In experimental studies and literature reports, researchers should report the specific operation steps of drug loading processes and evaluate specific loading and pharmacokinetic parameters in detail, to provide a truly valuable reference for the functional development of EV drug delivery.

### 5.6 EVs combine with scaffold

Scaffold materials provide a broader prospect for EVs applications. There are many advantages of combining EVs with scaffold in periodontal regeneration (130). To begin with, scaffold materials retain EVs in a specific location, especially in the periodontium. The multi-layered structure and the washout of saliva and gingival crevicular fluid can make those free EVs more easily to diffuse, thereby reducing their effectiveness. The application of scaffold materials can facilitate EVs’ retention in the periodontal pocket, preserve their bioactivity and reduce their risk in direct diffusion (131). Next, by encapsulating or carrying EVs in their ECM-like structure, scaffold materials might also control the concentration in local, achieve the sustained and controlled release *in vivo* and avoid the rapid clearance of EVs in circulation (92). Finally, this structure could provide the architectural restoration and biomechanical support for defective periodontal tissue (132). However, so far, the optimal solution for combining strategies still needs further study.

Hydrogel’s functions are different from the those of EVs in periodontal regeneration. Considering the biological mechanism, hydrogel is a common biological material in periodontal tissue regeneration engineering. It plays roles as scaffold, release-controlling material and ECM mimic. While EVs exert their functions by bioactive molecule delivery and information transport among cells. For example, EVs may well regulate bioactivities and metabolism of receptor cells. Those receptor cells, which take the role of “seed cells” and might be enhanced by EVs, rebuild a new tissue by their stimulated proliferation, migration and differentiation.

Many kinds of scaffold materials could be used as EV-carriers. Shen et al.’s (41) experiment used chitosan hydrogel for exosome-loading, Shi et al.’s (92) used gelatin hydrogel, Cui et al.’s (78) used GelMA hydrogel. Collagen has been applied in Tang et al.’s (92), Chew et al.’s (41) and Huang et al.’s (42) regenerative experiments, and it been proven to be beneficial for the final outcome. For example, Shen, Z. extracted exosomes from DPSCs. Incorporated into chitosan hydrogel, these exosomes facilitated the transition of macrophages from a pro-inflammatory to an anti-inflammatory phenotype, and accelerated healing of the alveolar bone and periodontal epithelium in periodontitis-afflicted mice (66). The theoretical reviews of scaffold materials for EVs can refer to articles of Ma et al. (133), Kasey S Leung et al. (134) and Wang et al. (73).

### 5.7 EVs' administration

Route of EV administration consists of local injection and systematic administration, which affects the efficiency of EV-based therapy.

First, injecting EVs is the simplest approach for researchers. In animal models, EVs are typically mixed with phosphate buffer solution and are directly injected into local tissue. This method may increase the bioavailability of EVs in complex periodontal tissue and circumvent the limitations of systemic administration. But this is a painful and invasive operation which may cause swelling, inflammation and tissue damage. Moreover, calculating appropriate dose of EVs, determining reasonable frequency of injection, controlling suitable concentrations in tissue, analyzing biological distribution and evaluating systematic clearance pose serious challenges to scientists. Despite these, the direct injection has become the most common way for its effectiveness and convenience according to the current literature. Numerous studies have demonstrated the efficacy of this strategy in periodontal treatment (67, 70, 84, 118, 127).

Secondly, through binding with biomaterials, EVs can be incorporated into periodontal tissue (135). For example, loaded onto a collagen sponge, human MSC exosomes were used to treat periodontal intrabony defects. Compared to the control group, rats with MSC exosomes showed improved periodontal defect repair, that is: enhanced new bone formation and promoted periodontal membranes regeneration, with increased cell infiltration and proliferation (41).

Thirdly, EVs can enter the body through the systemic route of administration, such as: intravenous administration, oral administration, etc. This approach has high clinical utility and eliminates the risk of local damage, and it is expected to target inflammatory tissue and exert therapeutic functions. However, studies have shown that systematically administered EVs accumulate rapidly in livers, spleens, lungs and kidneys. They are rapidly cleared in circulation, with insufficient concentrations in target tissue (136).

For example, Mahmoud Elashiry studied the relationship between the route of administration and the distribution in the body. Infused with radiolabeled IN-111, EXOs in intravenous route were slowly cleared in the mice’ bodies. These EXOs were absorbed into the systemic circulation such as spleen, but not to the injured maxillary site. Mice, using the local injection, showed 10-fold higher expression of radioactivity in inflamed lesion sites than the control group (112).

Although the potential to cross the physiological barriers renders EV promising biological agents, there are so many uncertainties in the administration process, such as short half-life, non-specific targeting and inefficiency of functional items. In the future, it is necessary to deal with these tackles, to add types of EVs, to increase more animal models, to make more rigorous design and operations, to establish more crucial parameters, to prevent off target effects and to provide more valuable evidence, for EVs’ application and transformation.

## 6 Challenges and prospects

EV-based treatment for periodontal tissue engineering is still in its infancy, but it is steadily growing and maturing. In fact, many challenges remain needed to be addressed (**Table 8**).

**Table 8:** Challenges and future suggestions.

	Challenges	Future Research Suggestions
EVs’ Biological mechanism		
Biogenesis	Heterogeneity of EV subpopulations; How do chemical and physical factors affect the biogenesis of evs?	Elucidating EV biogenesis
Cargo sorting	Unknow mechanisms that drive and regulate molecules entry into evs; Source of unknown active biomolecules	Elucidating precise mechanisms governing the sorting and integration of biological materials into evs
Release	Low yield of releasing	
Distribution	Unspecified EV targeting properties; Unclear biological distribution of EV *in vivo*; Unclear ability to cross multiple biological barriers, especially in pathologic conditions (e.g., increased vascular permeability)	Identifying homing/targeting specificities
Uptake	Unclear uptake mechanisms	Elucidating EV uptake mechanisms
Functional Effect	Effect of non-vesicular secretory factors (such as extracellular matrix proteins and cytokines)	More in-depth research on the biological effects as well as molecular mechanisms
EV engineering		
Harvesting samples and culture source sells	Source Selection; Nonstandard cultural protocols	Expanding available sources
EV separation and purification	Sophisticated isolation methods, poor purification, poor efficiency; poor reproducibility; low yield; the preparation processes affect the integrity and composition;	Optimize separation techniques; Identify and enrich the therapeutic cargo, minimize the ratio of other biomolecules
EV Characterization	Non-standard operation	Follow the provided standard guidelines and improve standardization and comparability
EV Modification and Targeting	Limited targeting ability; unsatisfactory distribution profile	Identify broader target receptors, leverage ligand–receptor interactions, minimized off-target effects, invent efficient strategies for uptake/fusion with target cells
EV Drug Loading	Low drug loading capability; compromised efficacy; low consistency; high cost; potential damage of EV and cargo	Rigorous methodologies
EV Assessment	Lack of assessment methodologies	Evaluate safety, efficacy, stability, biocompatibility, Pharmacokinetics, pharmacodynamics both *in vitro* and *in vivo*
EV and Scaffold		
EV administration	Low efficiency; Short half-life	
EVs' Functional Effect	Side-effects of other biomolecules in evs	
Others	Risk–benefit ratio; high production cost; standardized protocols for EV research	Summarize and establish standardized protocols
Clinical Translation		
	Large heterogeneity	Establish standardized protocols
	Absence of a placebo control	Set up reasonable control groups
	Absence of uniform quality control (QC) criteria	Alignment with national and international regulatory guidelines
	Systematical characterization of EV therapeutics	Develop new analytical tools; Evaluate safety and efficiency; Evaluate pharmacokinetics and pharmacodynamics; Analyses EV compositions and functions by precision methods; Analysis in Single-EV level
	Most of studies are small pilot trials	Large animal models (non-human primates)
	Differences between animal models and human	Carry on clinical trials
Industrial translation		
	Scalability and reproducibility	Scalable production procedure
	Lack of mature long-term storage approaches	Standardized procedure for EV storage and shipping
	Official standards for EV-based therapeutics	Standardizing critical quality attributes of EV
	Low cost-efficient outcome	
	Low production yield	

### 6.1 Research on the biological mechanism of EVs' periodontal treatment

Mechanisms in both the regeneration of periodontium and the biological functions EVs have long perplexed the researchers (137). Advanced knowledge on these processes is a prerequisite to develop effective therapies for periodontal regeneration. To the first, the exact mechanisms of biogenesis, sort, release and uptake of EVs have not been fully understood and require further investigation. It also mostly unknown how the EVs perform their functions in receptor cells and target tissue. A more comprehensive understanding will aid in developing suitable cultivation methods to alter EVs’ compositions and enhance their functions, to minimize off-target effects and improve targeted therapeutic approaches. Moreover, it would contribute to optimize their production process and enhance productivity (138). Secondly, although a conceptual model of periodontal regeneration has been proposed to guide experimental and clinical work, the specific mechanism of this process has not been firmly demonstrated. For example, the reason why seed cells are recruited can help to the regulation of migration and proliferation which improves therapeutic approaches in the end. The innate regenerative potential should be evaluated preciously for the selection of source sample of EVs. Therefore, researchers ought to investigate these scientific problems deeply, to provide more comprehensive insights into those controversial but important issues (139).

### 6.2 Optimize EV-based strategies

Lacking standardization of research processes impedes the development of EV-based strategies. Scientists could identify EVs formally when they begin their experiments. But there are some steps in the processes that people have no standard guideline. Their only way to conduct the experiment is to explore EVs based on their experience–protocols handed down from one generation of research assistants to another. This inconsistence complicates the comparing and referencing of EV-based results among different laboratories, putting obstacles in the way of collaborative work in this field. A systematic review and meta-analysis of preclinical trials illustrated that MSC-derived exosomes impressive potential for contributing to periodontal regeneration. But standardized and robust trials are required for the development of this technique (140). Therefore, to enhance the comparability and reproducibility of EV research, it is essential to explore a reasonable and standardized framework of EV-based therapy (141). And it is recommended that experiments should be designed, conducted and reported comprehensively according to guidelines for experimental knowledge bases and working process in the Minimal Information for Studies of Extracellular Vesicles (MISEV) of 2018 and 2024 (43, 116). A critical systematic review has been reported talking about of EV’s clinical trial. It highlighted the meticulous methodological reporting to enhance the successful clinical translation (142). To do so, the substitution of systematic protocols instead of personal work habits constitutes great options.

Other improvements rely on the safety and efficacy of EV engineering products. Researchers should focus on the systematic integration of research techniques, to improve the purity and yield, reduce the time cost and improve the efficacy of EV products in the future, as they are more suitable for periodontal application, as well as for clinical and industrial translation (143). More details could be learn from the systematic review of Van Delen etc. (144).

### 6.3 Explore EVs' clinical applications

A huge gap exists between the experimental research at present and the clinical requirements in the future. Firstly, absence of systematical characterization of EV therapies restricts the clinical transformation of EV. It is important to evaluate the safety and efficacy of the novel subcellular tool (145). Considered to enjoy high safety and low immunogenicity, EVs lack sufficient strong data and long-term evidence for these claims (143, 146). As the case stands, most of published reports remain small pilot trials. In its future work, the potential tumorigenicity and immunogenicity must be examined before clinical trials (147). As to efficacy, despite various attempts at the endogenous regeneration, these strategies often fail to demonstrate significant advantages in terms of the regenerative outcomes such as alveolar bone repair, periodontal ligament reconstruction and cementum regeneration. Further studies are required to develop new analytical tools, to evaluate safety and efficiency, to evaluate pharmacokinetics and pharmacodynamics, to analysis EV compositions and functions and to assess the functional effects of EV therapies. Secondly, inadequate uniform quality control (QC) criteria (17). Official research guidelines should provide standardized methods for more rigorous, more reproducible and more comparable research. In the scoping review of the literature of Clorinda Fusco in 2024, 40 studies about EVs as human therapies demonstrated that the large heterogeneity in EV experimental settings impeded the comparison across studies of the similar kind (143). Thirdly, there are regulatory challenges of EV-based strategies. Due to the high heterogeneity of natural EVs, complicated production processes, absent industrial specifications and lack of quality control guidelines, government regulatory is crucial to increase efficacy and promote safety (17). Next, most of the studies are small pilot trials (143), but clinical translation requires large animal model (non-human primates) experiments for more plausible conclusions. Furthermore, it is important for EV-based studies to set up reasonable control groups. Most of the current research literature lacks placebo controls, which are rigorous, especially for efficacy studies of biological agents.

### 6.4 Enhance EVs' industrial technology

There remains much to be done in EV manufacture to meet growing demands of industrial transformation (44). To the first, it’s a serious challenge to increase the scalability and reproducibility of EV production. Separation scalability, evaluation standardization, loading stability, modification reproducibility, standardization of functional experiments and mature of storage approaches are all technical problems to be overcome on the road to industrialization. Then, the low cost-efficient outcome undermines the confidence of researchers. In the future, close collaboration of biology, chemistry, engineering and periodontal medicine will be conducive to the progress of EV technology, thereby reducing the production cost, shortening the production time, improving the production efficiency and enhancing the therapeutic effect.

## 7 Conclusion

Collaborative efforts at EV-based treatment are bridging the gap between the ideal periodontium and the current outcome. In the future, inevitable integration of EV-based techniques and regenerative medicine may pave the way for the therapeutic development, and EVs indeed show considerable potential in the field of periodontology.

## Glossary

AMPK: Adenosine monophosphate-activated protein kinase
ADSCs: Adipose tissue-derived stem cells
BMMΦ: Bone marrow macrophages
BMMSCs: Bone marrow stem cells
BMP2: Bone morphogenetic protein-2
CCL2: C-C motif chemokine ligand 2
CXCR4: C-X-C motif chemokine receptor 4
DCs: Dendritic cells
DPSCs: Dental pulp stem cells
ECM: Extracellular matrix
MMP-8: Extracellular matrix metalloproteinase-8
MMP-9: Extracellular matrix metalloproteinase-9
EV: Extracellular vesicle
FGF-2: Fibroblast growth factor-2
GELNs: Ginger extracellular vesicle-like nanoparticles
GMSC: Gingival marrow stem cell
GTR: Guided tissue regeneration
Th1: Helper T type 1
Th2: Helper T type 2
Th17: Helper T type 2
HBP35: Heme-binding protein 35
HUVECs: Human umbilical vein endothelial cells
IGF-1: Insulin like growth factor-1
IL-10: Interleukin-10
IL-12: Interleukin-12
IL-1b: Interleukin-1b
IL-8: Interleukin-8
MVBs: Intracellular multivesicular bodies
ILVs: Intraluminal vesicles
LPS: Lipopolysaccharide
MSC: Mesenchymal stem cells
NOS: Nitric oxide synthase
NF-κB: Nuclear transcription factor-κB
OPG: Osteoprotegerin
PDLSCs: Periodontal ligament stem cells
PDGF-BB: Platelet-derived growth factor-BB
PGE-2: Prostaglandin E2
RANK: Receptor activator for nuclear factor-κ
RANKL: Receptor-activator of nuclear factor kappa beta ligand
SHEDs: Stem cells from human exfoliated decid-uous teeth
TRAP: Tartrate resistant acid phosphatase
3D: Three-dimensional
TGF-β: Transforming growth factor-β
Treg: T-regulatory cells.
TCP: Tricalcium phosphate
TNF-a: Tumor necrosis factor-α
